# Profiles of Innovative Behaviour Among Community Nurses and Associated Factors: A Cross‐Sectional Study

**DOI:** 10.1002/nop2.70819

**Published:** 2026-09-21

**Authors:** Shengcai Zhu, Jiaojiao Hu, Beirong Mo, Junze Yan, Kunqi Ma, Yumei Liao, Li Wang

**Affiliations:** ^1^ Department of Nephrology Shenzhen Nanshan People's Hospital Shenzhen China; ^2^ Shenzhen Nanshan Medical Group Headquarters Shenzhen China; ^3^ Queen Mary College Nanchang University Nanchang China; ^4^ Shenzhen Xinhua Hospital Shenzhen China

**Keywords:** community health nursing, innovative behaviour, latent profile analysis, nursing management, person‐centred analysis, professional identity, telehealth readiness

## Abstract

**Background:**

Innovative behaviour is pivotal for advancing community nursing, yet it is predominantly examined as a monolithic, variable‐centred construct. This traditional approach overlooks the inherent heterogeneity in how individual nurses innovate. A person‐centred paradigm is therefore crucial to identify distinct subgroups of innovators within the workforce.

**Aims:**

This study aimed to identify distinct latent profiles of innovative behaviour among Chinese community nurses and examine how individual antecedents (such as professional identity and telehealth readiness) and organizational antecedents (such as perceived support and knowledge sharing) are associated with profile membership.

**Design:**

A cross‐sectional descriptive survey design was employed.

**Methods:**

A cross‐sectional survey was conducted with 395 community nurses recruited via convenience sampling from 87 community health centres in Shenzhen, China. Participants completed validated scales including the nurse innovative behaviour scale (NIBS), the professional identity Scale, the telehealth readiness assessment tools—Chinese, and the Perceived Organizational Support Scale, along with demographic and key work‐related antecedents including training access and managerial knowledge sharing. Latent profile analysis (LPA) was conducted using the dimensions of the NIBS as indicators. Multinomial logistic regression was employed to analyse the associated factors of profile membership.

**Results:**

A two‐profile solution provided the best model fit. Profile 1 was labelled the Moderate Innovation Profile (50.4%) and was characterized by a consistent, modest pattern of innovation activity, whereas Profile 2 was labelled the High Innovation Profile (49.6%) and exhibited a proactive, high‐level pattern across all dimensions. Multinomial logistic regression with the Moderate Innovation Profile as the reference revealed that higher education, prior general hospital experience, research participation, access to innovation training, managerial knowledge sharing, stronger professional identity and higher telehealth readiness were significant predictors for membership in the High Innovation Profile.

**Conclusion:**

Community nurses' innovative behaviour is not uniform but manifests in two distinct profiles. This finding challenges the traditional one‐size‐fits‐all approach to nursing management. The results provide actionable insights indicating that interventions should be tailored to key antecedents. To cultivate the High Innovation Profile, healthcare organizations should prioritize enhancing digital and telehealth readiness and promoting managerial knowledge sharing, alongside fostering research engagement.

## 1 Introduction

Against the backdrop of transformative challenges in global healthcare, innovation is a critical driving force for advancing the nursing discipline and ensuring patient safety (Ouyang and Lu 2021; Zhang et al. 2022). Nurse innovative behaviour (IB) represents a multi‐stage process where staff develop and apply new clinical methods after gaining organizational support (Lv et al. 2021; Ma et al. 2025). This behaviour serves as a powerful engine for sustaining the profession and consistently enhancing care quality (Liu and Li 2015; Yan and Li 2021; Sha et al. 2023). As frontline providers with the closest patient contact, nurses are uniquely positioned to identify clinical problems and thereby initiate innovation (Gordon et al. 2022). Ultimately, IB involves generating and implementing novel ideas to optimize individual, team, or organizational performance (Akkoç et al. 2022; Wu et al. 2025). Of particular importance is proactive IB, a voluntary action aimed at seizing future opportunities and preemptively solving potential clinical problems (Abdelwahab Ibrahim El‐Sayed et al. 2024). This proactive stance often yields superior, high‐quality outcomes, thereby continually enhancing a team's overall innovative capacity (Ma et al. 2023). Therefore, understanding the nuances of IB is essential for fostering a resilient and adaptive nursing workforce.

The global trends of population ageing and rising chronic disease burdens have precipitated a fundamental shift in healthcare delivery from acute hospital‐centric models to community‐based settings. This transition places unprecedented demands on community health services, which must now manage higher patient acuity, complex co‐morbidities and significant public health mandates. In this context, fostering innovation is an international consensus, which is explicitly echoed by China's National Nursing Development Plan (2021–2025) (National Health Commission of the PRC 2022). Among all nursing fields, community nursing demands faster and more frequent innovation due to its unique service models and complex work environments. Unlike their hospital counterparts, community nurses often work with greater autonomy, face stricter resource limitations, and must navigate complex social and family dynamics. As the core of this workforce, community nurses' innovation is manifested not only in technological updates but also in the transformation of service philosophies and care models. Therefore, fostering community nurse innovation is practically essential to optimize frontline care delivery and ensure patient safety in primary healthcare settings.

However, a critical gap pervades the existing literature: IB is predominantly treated as a monolithic, homogeneous construct, which effectively masks the significant heterogeneity that exists within the nursing population. However, a significant empirical gap remains: existing research predominantly treats innovative behaviour as a monolithic construct, completely masking the inherent heterogeneity and distinct individual patterns within the nursing workforce (Collins and Lanza 2009). This perspective fails to clarify why nurses with similar demographic profiles exhibit disparate patterns of innovation, representing a major empirical omission that obscures the phenomenon's complexity. For instance, some nurses may excel at idea generation but falter during implementation, while others may be adept adopters of existing innovations but rarely propose new ones. A single mean score fails to capture these nuanced profiles. This ‘one‐size‐fits‐all’ assumption leads to inefficient, non‐targeted managerial interventions, highlighting an urgent practical gap in tailored nursing administration. To overcome this, a person‐centred approach is required to systematically identify the distinct levels and patterns of innovation engagement that exist within the workforce.

Latent profile analysis (LPA) is a sophisticated, person‐centred statistical method that classifies individuals into distinct subgroups based on their response patterns on a set of observed variables, thereby characterizing the features of each latent class (Zuo 2025). By capturing the unique configurations of innovation dimensions within individuals, this person‐centred approach extends the existing nursing literature beyond the limitations of sample‐wide averages, offering a more nuanced framework for nursing scholarship and workforce development. However, merely identifying profiles is insufficient; we must also understand the antecedents that predict why nurses cluster into these distinct groups. To guide this investigation, we adopt the model of individual innovative behaviour developed by Scott and Bruce (Scott and Bruce 1994). This foundational model posits that individual innovative behaviour is an outcome of the interplay between individual characteristics and the surrounding work environment. It suggests that innovation flourishes when motivated individuals, who possess the requisite skills, are actively supported by their leaders and the broader organizational climate. Building on this logic, our study conceptualizes this multi‐level system by examining key variables at each nexus. We begin with the individual, hypothesizing that professional identity (PI) provides the core motivational drive to challenge the status quo, while telehealth readiness (TR) represents the critical capability required in the specific context of modern community nursing. This individual potential, however, must be activated by leadership. We posit that this activation occurs through managerial knowledge sharing, a key behaviour that provides the task‐relevant information needed to move ideas forward. Finally, this entire process is embedded within the broader organizational context, which we assess via the general psychological climate of perceived organizational support (POS) and the specific, tangible resource of access to departmental innovation training. This multi‐level framework provides a robust theoretical foundation for exploring the complex determinants of innovation profiles.

This study aims to identify the latent profiles of IB among community nurses in China and explore how the identified multi‐level theoretical antecedents predict membership in these distinct subgroups. This study also exploratorily examines the role of other key demographic and work‐related characteristics (such as higher education, general hospital experience and research participation) in differentiating these profiles. Theoretically, this study advances nursing science by revealing how individual characteristics, such as telehealth readiness, and environmental support shape distinct innovation profiles among community nurses. Practically, it provides empirical evidence for nursing managers to design tailored support and training strategies based on the specific features of each profile.

## 2 Methods

### 2.1 Study Design and Participants

This study utilized a cross‐sectional design and followed the STROBE reporting guidelines. A convenience sampling method was employed to recruit participants from 87 public community health service centres affiliated with a large tertiary hospital in southern China between August and September 2025. These centres cover a diverse range of urban and suburban neighbourhoods, providing a wide range of demographic characteristics from the local nursing workforce. The inclusion criteria were as follows: (1) full‐time nurses officially registered at the participating centres; (2) a minimum of 1 year of continuous clinical work experience to ensure familiarity with community health workflows; and (3) provided informed consent and voluntarily agreed to participate. The exclusion criteria were nurses who were on extended leave (more than 3 months, e.g., maternity or sick leave), in rotational programs from other hospitals, or undergoing advanced training away from their primary clinical site during the study period. Previous research suggests a minimum sample size of 300 for LPA studies (Ferguson et al. 2020). The final effective sample size for this study was 395, exceeding the recommended threshold for robust profile identification. Ethical approval for this study was granted by the Ethics Committee of the participating hospital (Approval No. ky‐2025‐062301). The research was conducted in compliance with the Declaration of Helsinki, and all participants provided written informed consent prior to data collection.

### 2.2 Measures

To evaluate the factors associated with community nurses' innovative behaviour comprehensively, the measurement protocol assessed three distinct domains: internal psychological evaluation of professional identity, technical capacity of telehealth readiness and external workplace resources of perceived organizational support.

#### 2.2.1 Demographic Questionnaire

A self‐designed questionnaire, based on a review of relevant literature, was used to collect data on participants' characteristics, including gender, age (years), marital status, highest educational attainment, professional title, years of work experience, employment type, prior work experience in a general hospital, number of night shifts per month, average monthly income (including salary and bonuses), participation in research projects, availability of departmental innovation training and whether their manager shares research knowledge.

#### 2.2.2 Nurse Innovative Behaviour Scale (NIBS)

The NIBS, developed by Bao et al. (2012), is a 10‐item scale that measures three dimensions of innovative behaviour: idea generation, support seeking, and idea realization. Responses are rated on a 5‐point Likert scale, ranging from 1 (Never) to 5 (Very Frequently), where higher scores indicate a greater propensity for innovative behaviour. The original scale demonstrated good reliability, with a Cronbach's *α* of 0.879 for the total scale and between 0.746 and 0.870 for its subscales. In this study, the Cronbach's *α* for the total scale was 0.951.

#### 2.2.3 Professional Identity Scale (PIS)

The PIS, developed by Liu (Liu 2009), is a 30‐item scale consisting of five dimensions: professional cognitive evaluation, professional social skills, professional social support, career frustration, and professional self‐reflection. Participants rate items on a 5‐point Likert scale from 1 (Strongly Disagree) to 5 (Strongly Agree). Higher scores reflect a stronger sense of professional identity. The original scale showed excellent reliability, with a Cronbach's *α* of 0.938 for the total scale and ranging from 0.720 to 0.910 for its subscales. In this study, the Cronbach's *α* for the total scale was 0.986.

#### 2.2.4 Telehealth Readiness Assessment Tools—Chinese (TRAT‐C)

The TRAT‐C is the Chinese version of a scale originally developed by Jennett et al. (2003) and subsequently translated and adapted by Liu et al. (2020). This 17‐item instrument assesses three dimensions of readiness: core readiness, engagement readiness, and structural readiness. Items are rated on a 5‐point Likert scale (1 = ‘Strongly Disagree’ to 5 = ‘Strongly Agree’). The TRAT‐C has demonstrated good reliability and validity, with a Cronbach's *α* of 0.861 and a content validity index ranging from 0.88 to 1.00. In this study, the scale's Cronbach's *α* was 0.937.

#### 2.2.5 Perceived Organizational Support Scale (POSS)

This study utilized a version of the POS scale developed by Chen (Chen 2006) and later adapted for the nursing context by Zuo and Yang (2009). The scale is widely used in nursing research and comprises 13 items across two dimensions: emotional support and instrumental support. A 5‐point Likert scale (1 = ‘Strongly Disagree’ to 5 = ‘Strongly Agree’) is used, with higher scores indicating a greater level of perceived organizational support. The scale has a reported Cronbach's *α* of 0.921. In this study, the Cronbach's *α* for the total scale was 0.979.

### 2.3 Data Collection Procedures

Data were collected online via a unique survey link and a corresponding QR code generated by the Wenjuanxing platform. The principal investigator first contacted the nurse managers of the participating community health centres to explain the study's objectives, target population and survey methodology. Upon receiving their approval, the survey link and QR code were distributed to the nurse managers via WeChat, who then disseminated them to eligible nurses during staff meetings or via internal work groups. An electronic informed consent form was presented on the first page of the survey, and participants voluntarily agreed to participate by clicking ‘agree’ before proceeding. Participation was completely voluntary and anonymous. All completed surveys were submitted directly to the online platform without any institutional tracking, ensuring that individual responses remained entirely inaccessible to nurse managers or supervisors. To ensure data integrity, the online system was configured with mandatory response fields, prompting participants to complete any missed items before submission. To prevent duplicate entries, the system was set to allow only one submission per IP address and user account. To guarantee data quality, questionnaires were excluded from the final analysis if they were completed in less than 3 min, contained contradictory responses, or exhibited straight‐lining (e.g., selecting the same option for 10 or more consecutive items). Data were exported directly from the electronic survey platform for statistical analysis. Incomplete records or invalid questionnaires, such as those failing to meet the minimum response time threshold established in the pretest were strictly excluded from the final analysis to ensure data quality.

### 2.4 Statistical Analysis

All statistical analyses were conducted using SPSS version 27.0 and Mplus version 8.3. Prior to the primary analysis, a full collinearity assessment and Harmans single factor test were performed to examine common method bias. Descriptive statistics were used to summarize the sample characteristics. Continuous variables were presented as means and standard deviations, while categorical variables were presented as frequencies and percentages.

An LPA was performed using Mplus to identify distinct subgroups of innovative behaviour based on shared response patterns. Following the default parameter specifications in Mplus, the models were estimated under the assumption of local independence, where indicator variances were constrained to be equal across classes and all within‐class covariances were fixed to 0. The scores from the three dimensions of the NIBS were used as the indicator variables. To ensure the global maximum likelihood was reached and to avoid local maxima, we utilized the default random starts involving 20 initial stage starts and 4 final stage optimizations, and thoroughly verified that the best log‐likelihood value was successfully and consistently replicated for each multi‐class model. The Robust Maximum Likelihood (MLR) estimator was employed to account for any non‐normality in the data. A series of LPA models were estimated, starting with a one‐profile model (*k* = 1) and sequentially adding profiles (*k* = 2, *k* = 3, etc.). The optimal number of profiles was determined by evaluating several model fit indices in accordance with established guidelines (Cang et al. 2026; Wu et al. 2026; Zhang et al. 2023). These criteria included: the Akaike Information Criterion (AIC), the Bayesian Information Criterion (BIC) and the sample‐size adjusted BIC (aBIC), with lower values indicating a better model fit; high classification quality as indicated by entropy values (closer to 1); and the Lo–Mendell–Rubin likelihood ratio test (LMR‐LRT) and the Bootstrapped Likelihood Ratio Test (BLRT), where a significant *p*‐value (*p* < 0.05) suggests the *k*‐profile model provides a significantly better fit than the *k*‐1 profile model. Finally, the interpretability and parsimony of the model were considered, with a minimum class‐size criterion of 5% prespecified to ensure that any retained profile possessed sufficient statistical power and practical distinctiveness.

Once the optimal profile solution was determined, differences in demographic characteristics and antecedent variables across the profiles were examined. Chi‐square (*χ* ^2^) tests were used for categorical variables. For continuous variables, independent samples *t*‐tests were employed when data met the assumptions of normality and homogeneity of variance, and the non‐parametric Mann Whitney *U* test was performed otherwise. Finally, multinomial logistic regression was performed using SPSS to examine the predictors of profile membership. In this model, ordinal variables, such as education level and professional title, were treated as nominal variables and entered as dummy variables to calculate specific odds ratios for each individual category relative to the reference group. For all analyses, statistical significance was set at a two‐sided *p* < 0.05.

## 3 Results

### 3.1 Common Method Bias Test

As the data in this study were collected via self‐report measures from a single source, Harman's single‐factor test was employed to assess the potential for common method bias (Shi 2019). Prior to analysis, procedural remedies, such as guaranteeing anonymity and clarifying that there were no right or wrong answers, were strictly implemented during data collection to minimize social desirability bias. All relevant items were subjected to an unrotated exploratory factor analysis. The results revealed eight factors with eigenvalues greater than 1 (detailed in Table 1). The first factor accounted for 48.691% of the total variance, which is below the recommended 50% threshold. Therefore, while common method bias does not account for the majority of the variance, its potential influence cannot be entirely ruled out and should be considered when interpreting the findings. To further evaluate the rigour of the data and potential common method variance, a full collinearity assessment was performed for all predictors. The variance inflation factors for all variables ranged from 1.032 to 2.919, which are all well below the conservative threshold of 3.0. These findings provide additional statistical evidence that multicollinearity is absent, and common method bias is unlikely to be a major threat to the validity of the results, though the findings should be interpreted with caution.

**TABLE 1:** Common factors with eigenvalues greater than 1.

Component	Total	Initial eigenvalue variance percentage	Cumulative %	Total	Extracted load square variance percentage	Cumulative %
1	34.083	48.691	48.691	34.083	48.691	48.691
2	7.986	11.408	60.099	7.986	11.408	60.099
3	3.566	5.095	65.193	3.566	5.095	65.193
4	3.039	4.341	69.534	3.039	4.341	69.534
5	2.195	3.136	72.670	2.195	3.136	72.670
6	1.768	2.525	75.195	1.768	2.525	75.195
7	1.099	1.570	76.766	1.099	1.570	76.766
8	1.035	1.479	78.244	1.035	1.479	78.244

### 3.2 Participant Characteristics

A total of 420 eligible community nurses were invited to participate via electronic links and QR codes distributed through direct nurse managers, and 395 completed valid questionnaires, yielding an effective response rate of 94.1%. The majority of participants were female (93.9%), held a bachelor's degree (64.1%) and had a senior nurse professional title (31.6%). Detailed demographic and work‐related characteristics of the sample are presented in Table 2.

**TABLE 2:** Socio‐demographic and professional characteristics of participants by latent profile.

Variable	*N* (*n* = 395)	Practice guardians	Professional leaders	Test statistic	*p*
Gender					
Male	1 (0.3%)	0 (0%)	1 (0.3%)	1.404	0.236^c^
Female	394 (99.7%)	199 (50.4%)	195 (49.3%)		
Age (years)					
< 25	16 (4.1%)	9 (2.3%)	7 (1.8%)	8.562	0.036^a^
25–34	92 (23.3%)	42 (10.6%)	50 (12.7%)		
35–44	208 (52.7%)	97 (24.6%)	111 (28.1%)		
≥ 45	79 (20.0%)	51 (12.9%)	28 (7.1%)		
Marital status					
Single	64 (16.2%)	30 (7.6%)	34 (8.6%)	0.417	0.812^a^
Married	318 (80.5%)	162 (41.0%)	158 (39.5%)		
Divorced/Widowed	13 (3.3%)	7 (1.8%)	6 (1.5%)		
Highest Educational Attainment					
Technical Secondary School/Vocational School	37 (9.4%)	19 (4.8%)	18 (4.6%)	14.061	0.003^a^
Associate Degree/Diploma	81 (20.5%)	54 (13.7%)	27 (6.8%)		
Bachelor's Degree	253 (64.1%)	119 (30.1%)	134 (33.9%)		
Master's Degree or above	24 (6.1%)	7 (1.8%)	17 (4.3%)		
Professional title					
Nurse	19 (4.8%)	6 (1.5%)	13 (3.3%)	16.333	< 0.001^a^
Senior Nurse	125 (31.6%)	80 (20.3%)	45 (11.4%)		
Nurse‐in‐charge	232 (58.7%)	107 (27.1%)	125 (31.6%)		
Associate Chief Nurse or above	19 (4.8%)	6 (1.5%)	13 (3.3%)		
Years of work experience (years)					
< 5	23 (5.8%)	7 (1.8%)	16 (4.1%)	13.809	0.003^a^
5–9	54 (13.7%)	29 (7.3%)	25 (6.3%)		
10–19	156 (39.5%)	66 (16.7%)	90 (22.8%)		
> 19	162 (41.0%)	97 (24.6%)	65 (16.5%)		
Employment type					
Temporary employment	33 (8.4%)	17 (4.3%)	16 (4.1%)	0.456	0.796^a^
Contract‐based	314 (79.5%)	160 (40.5%)	154 (39.0%)		
Tenured/Permanent staff	48 (12.2%)	22 (5.6%)	26 (6.6%)		
Have you ever worked in a general hospital?					
Yes	287 (72.7%)	116 (29.4%)	171 (43.3%)	41.668	< 0.001^a^
No	108 (27.3%)	83 (21.0%)	25 (6.3%)		
Number of night shifts per month					
0	133 (33.7%)	63 (15.9%)	70 (17.7%)	2.766	0.429^a^
1–3	56 (14.2%)	25 (6.3%)	31 (7.8%)		
4–6	62 (15.6%)	31 (7.8%)	31 (7.8%)		
> 6	144 (36.5%)	80 (20.3%)	64 (16.2%)		
Average monthly income (including salary and bonuses)					
≤ 5000	7 (1.8%)	2 (0.5%)	5 (1.3%)	3.175	0.529^a^
5001–10,000	104 (26.3%)	48 (12.2%)	56 (14.2%)		
10,001–15,000	222 (56.2%)	118 (29.9%)	104 (26.3%)		
15,001–20,000	48 (12.2%)	23 (5.8%)	25 (6.3%)		
≥ 20,001	14 (3.5%)	8 (2.0%)	6 (1.5%)		
Have you participated in research projects?					
Yes	94 (23.8%)	20 (5.1%)	74 (18.7%)	41.795	< 0.001^a^
No	301 (76.2%)	179 (45.3%)	122 (30.9%)		
Does your department offer innovation training?					
Yes	176 (44.6%)	39 (9.9%)	137 (34.7%)	101.131	< 0.001^a^
No	219 (55.4%)	160 (40.5%)	59 (14.9%)		
Does your manager share research knowledge?					
Yes	198 (50.1%)	61 (15.4%)	137 (34.7%)	60.833	< 0.001^a^
No	197 (49.9%)	138 (35.0%)	59 (14.9%)		
POS score ($\bar{X}$ *± s*)	395	46.67 ± 9.88	52.24 ± 8.86	−6.200	< 0.001^b^
PIS score ($\bar{X}$ *± s*)	395	107.78 ± 20.28	122.25 ± 16.44	−7.170	< 0.001^b^
TRAT‐C score($\bar{X}$ *± s*)	395	55.23 ± 9.64	64.46 ± 9.79	−9.068	< 0.001^b^

^a^ *χ* ^2^ value.

^b^ *Z* value (Mann–Whitney *U* test).

^c^ Fisher's exact test.

### 3.3 Results of the Latent Profile Analysis for Innovative Behaviour

#### 3.3.1 Model Selection

LPA models with one to four latent profiles were fitted to the data using the three dimensional scores of the NIBS as indicator variables. As shown in Table 3, the AIC, BIC and aBIC values generally decreased as the number of profiles increased. However, for the three‐ and four‐profile models, the LMR‐LRT was non‐significant (*p* > 0.05), suggesting that these models did not provide a significantly better fit than the models with one fewer profile. Consequently, the two‐profile model was selected as the optimal solution. This model was supported by its combination of relatively low AIC, BIC and aBIC values, significant LMR‐LRT and BLRT results (*p* < 0.05) and a high Entropy value (0.934). The average posterior probabilities for profile membership were excellent (ranging from 0.980 to 0.987), indicating high classification accuracy.

**TABLE 3:** Fit indices for latent profile models with one to four profiles (*n* = 395).

Number of profiles	AIC	BIC	aBIC	LMR‐LRT (*p*)	BLRT (*p*)	Entropy	Class proportions
1	5325.987	5349.860	5330.822	—	—	—	—
2	4784.193	4823.982	4792.252	< 0.001	< 0.001	0.934	50.4%/49.6%
3	4611.208	4666.912	4622.490	0.0894	< 0.001	0.959	46.1%/49.1%/4.8%
4	4236.576	4308.196	4251.082	0.4945	< 0.001	0.994	7.7%/40.5%/46.6%/5.3%

Abbreviations: aBIC, adjusted Bayesian Information Criterion; AIC, Akaike Information Criterion; BIC, Bayesian Information Criterion; BLRT, bootstrapped likelihood ratio test; LMR‐LRT, Lo–Mendell–Rubin likelihood ratio test.

#### 3.3.2 Profile Characteristics

**FIGURE 1:**
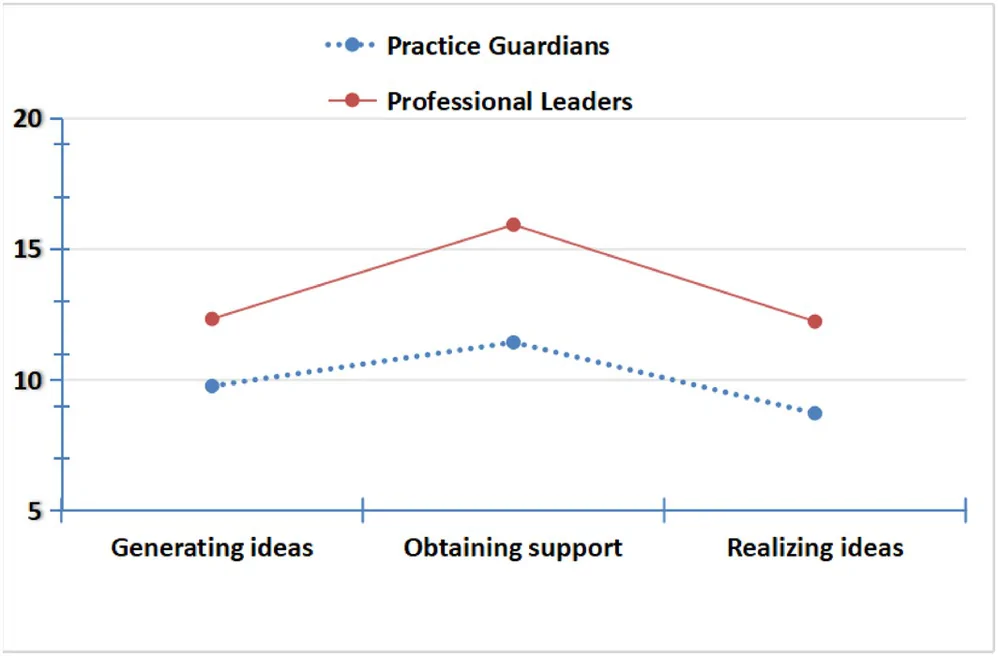
Plot of the mean scores on innovative behaviour dimensions across the two latent profiles.

Based on this two‐profile solution, the profiles were named according to their distinct patterns on the NIBS dimensions (Figure 1): Profile 1 was labelled ‘Moderate Innovation Profile’ (*n* = 199, 50.4%), characterized by a consistent, modest pattern of innovation activity across all dimensions. Profile 2 was labelled ‘High Innovation Profile’ (*n* = 196, 49.6%), characterized by a proactive, high‐level pattern across all dimensions.

### 3.4 Univariate Analysis of Profile Membership

The results of the univariate analyses (presented in Table 2) revealed that there were statistically significant differences between the two profiles on the variables of age, highest educational attainment, professional title, years of work experience, prior experience working in a general hospital, participation in research projects, availability of departmental innovation training and whether their manager shares research knowledge (all *p* < 0.05).

### 3.5 Multinomial Logistic Regression Analysis of Profile Predictors

To identify the predictors of profile membership, a multinomial logistic regression analysis was conducted. The latent profile variable was entered as the outcome, with the ‘Moderate Innovation Profile’ profile serving as the reference group. The variables that were found to be significant in the univariate analyses were entered as predictors. For the analysis, the continuous variables (PI, TR and POS) were entered as measured. The coding scheme for the categorical predictors is detailed in Table 4.

**TABLE 4:** Assignment of independent variables.

Variable	Case of assignment
Gender	1 = Male, 2 = Female
Age (years)	1 = < 25, 2 = 25–34, 3 = 35–44, 4 = ≥ 45
Marital status	1 = Single, 2 = Married, 3 = Divorced/Widowed
Highest educational attainment	1 = Technical Secondary School/Vocational School, 2 = Associate Degree/Diploma, 3 = Bachelor's Degree, 4 = Master's Degree or above
Professional title	1 = Nurse, 2 = Senior Nurse, 3 = Nurse‐in‐charge, 4 = Associate Chief Nurse or above
Years of work experience (years)	1 = < 5, 2 = 5–9, 3 = 10–19, 4 = > 19
Employment type	1 = Temporary employment, 2 = Contract‐based, 3 = Tenured/Permanent staff
Have you ever worked in a general hospital?	1 = Yes, 2 = No
Number of night shifts per month	1 = 0, 2 = 1–3, 3 = 4–6, 4 = > 6
Average monthly income (including salary and bonuses)	1 = ≤ 5000, 2 = 5001–10,000, 3 = 10,001–15,000, 4 = 15,001–20,000, 5 = ≥ 20,001
Have you participated in research projects?	1 = Yes, 2 = No
Does your department offer innovation training?	1 = Yes, 2 = No
Does your manager share research knowledge?	1 = Yes, 2 = No

The final results of the regression analysis are presented in Table 5. Prior general hospital experience (OR = 5.144, *p* < 0.001) and participation in research projects (OR = 4.173, *p* < 0.001) were associated with a higher likelihood of being in the ‘High Innovation Profile’ profile. Regarding organizational factors, access to innovation training (OR = 6.203, *p* < 0.001) and managerial knowledge sharing (OR = 2.568, *p* < 0.003) were significant predictors. Finally, higher scores on PI (OR = 1.025, *p* < 0.045) and TR (OR = 1.077, *p* < 0.001) were also significantly associated with membership in the ‘High Innovation Profile’ profile, while POS was not (*p* = 0.371).

**TABLE 5:** Multinomial logistic regression analysis of predictors for latent profiles of innovative behaviour.

Variable	*β*	SE	Wald χ^2^	Exp (*B*)	95% CI	*p*
Age (years)						
< 25	−0.346	1.069	0.105	0.707	0.087 ~ 5.753	0.746
25–34	0.514	0.712	0.52	1.672	0.414 ~ 6.755	0.471
35–44	0.724	0.434	2.782	2.063	0.881 ~ 4.829	0.095
Highest educational attainment						
Technical Secondary School/Vocational School	−1.733	0.859	4.074	0.177	0.033 ~ 0.951	0.044
Associate Degree/Diploma	−1.746	0.781	5.003	0.174	0.038 ~ 0.806	0.025
Bachelor's Degree	−1.106	0.708	2.442	0.331	0.083 ~ 1.325	0.118
Professional title						
Nurse	0.625	1.019	0.376	1.868	0.254 ~ 13.756	0.54
Senior nurse	−0.786	0.757	1.079	0.455	0.103 ~ 2.009	0.299
Nurse‐in‐charge	0.641	0.71	0.813	1.898	0.472 ~ 7.638	0.367
Years of work experience (years)						
< 5	1.527	0.908	2.829	4.605	0.777 ~ 27.303	0.093
5–9	0.319	0.771	0.171	1.376	0.304 ~ 6.233	0.679
10–19	0.4	0.375	1.138	1.491	0.716 ~ 3.108	0.286
Have you ever worked in a general hospital?						
Yes	1.638	0.379	18.703	5.144	2.449 ~ 10.807	0.001
Have you participated in research projects?						
Yes	1.429	0.374	14.582	4.173	2.005 ~ 8.689	0.001
Does your department offer innovation training?						
Yes	1.825	0.341	28.623	6.203	3.178 ~ 12.104	0.001
Does your manager share research knowledge?						
Yes	0.943	0.321	8.621	2.568	1.368 ~ 4.819	0.003
POS score	−0.021	0.023	0.802	0.98	0.936 ~ 1.025	0.371
PIS score	0.024	0.012	4.017	1.025	1.001 ~ 1.049	0.045
TRAT‐C score	0.074	0.017	17.937	1.077	1.04 ~ 1.114	0.001

*Note:* The ‘Moderate Innovation Profile’ profile was the reference category for the analysis. For categorical predictors, the reference categories are: age ≥ 45 years, master's degree or above, Associate Chief Nurse or above, and years of work experience > 19 years. For all binary variables, ‘No’ is the reference category used as the baseline for comparison.

Abbreviations: *β*, standardized coefficient; CI, confidence interval; Exp(B), odds ratio; PIS, Professional Identity Scale; POS, perceived organizational support; SE, standard error; TRAT‐C, Telehealth Readiness Assessment Tools‐Chinese.; Wald *χ* ^2^, Wald chi‐squared.

## 4 Discussion

This study applies a person‐centred approach to understand innovation among community nurses identifying a Moderate Innovation Profile and a High Innovation Profile. While traditional variable‐centred methods provide useful aggregate scores, they fail to capture the distinct levels of innovation within a population (Conte and Harmata 2023). The identification of a Moderate Innovation Profile and a High Innovation Profile provides a more nuanced view of innovation than a monolithic linear scale (Zhang et al. 2024). Although these profiles differ primarily in overall level rather than shape, this distinction suggests that innovation is a cumulative capacity dependent on resources rather than a specialized trait. This core finding suggests that cultivating innovation requires a more nuanced re‐evaluation, moving beyond universal incentives and toward precision empowerment.

The near‐equal distribution of these groups in our sample highlights a potential level‐based heterogeneity, suggesting that a single uniform management strategy may not be fully effective (Hassmiller and Wakefield 2022). The near‐equal distribution of these groups confirms a level‐based heterogeneity, which implies that any single management strategy is destined to fail (Slåtten et al. 2023).

Our regression model indicates this differentiation is not random, but is driven by a complex interplay of factors that align well with the ‘model of individual innovative behavior’ proposed by Scott and Bruce (Scott and Bruce 1994). The accumulation of ‘professional capital’ emerged as a foundational predictor. Higher educational attainment was a significant factor, which aligns with research identifying advanced academic backgrounds as a key antecedent for innovative work behaviour in nurses (Baig et al. 2022). Access to innovation training with an odds ratio of 6.203 and prior general hospital experience with an odds ratio of 5.144 emerged as the most potent predictors of high innovation profile membership. These large effect sizes demonstrate that structured training and diverse clinical exposure are dominant catalysts that multiply the likelihood of high‐level innovative behaviour by more than six and five times, respectively (Mendívil‐Pérez et al. 2025). Moreover, the organizational environment plays a crucial activation role. Our results offer a crucial insight by distinguishing between generic and specific support. Generic Perceived Organizational Support (POS) was not a significant predictor (*p* = 0.371), whereas specific, empowering actions—managerial knowledge sharing (OR = 2.568) and the aforementioned innovation training—were highly significant. This indicates that broad organizational support fails to translate into proactive outcomes, whereas targeted knowledge sharing by direct supervisors more than doubles the odds of driving nurses into the high innovation group (Gharajeh‐Alamdari et al. 2025). Internal motivation factors, including professional identity (OR = 1.025) and telehealth readiness (OR = 1.077), were also statistically significant predictors of membership in the high innovation group (Wang et al. 2022; Bober et al. 2024). However, it is worth noting that the magnitude of these single‐unit effect sizes is relatively modest compared to potent categorical environmental catalysts like structured training. This indicates that while these continuous internal traits have a statistically significant impact, their practical contribution manifests as a small, incremental benefit for each one‐point increase on the scales. Nonetheless, over a broader distribution, digital readiness serves as a channel allowing community nurses to transcend local resource limitations and access a broader ecosystem of innovative practices.

These findings call for a strategic shift from static management to building a dynamic talent development pipeline. The goal should not be to fix nurses in their roles but to establish a developmental ladder that allows for talent mobility. For ‘Moderate Innovation Profile’, whom our model suggests may lack the specific experiential capital (OR = 5.144) and training capital (OR = 6.203) of their peers, the strategy must be foundational empowerment. This aims to directly address the identified gaps through quality improvement training and structured continuing professional development (Vázquez‐Calatayud et al. 2021). Furthermore, establishing structured mentorship programs that pair ‘Moderate Innovation Profile’ with experienced innovators can accelerate this skill acquisition (Gularte‐Rinaldo et al. 2023). This empowerment has a dual purpose: helping ‘Moderate Innovation Profile’ excel in their current roles, while also offering a voluntary path for high‐potential individuals to gain the skills needed to become ‘High Innovation Profile’. Conversely, for ‘High Innovation Profile’, who already possess high capital and the internal drive evidenced by high PI (OR = 1.025) and TR (OR = 1.077) scores, the strategy must be catalytic empowerment. This shifts from building capital to unleashing it, and crucially, appoints them as the internal engine for this talent pipeline. We recommend that administrators formally leverage individuals within the High Innovation Profile as quality improvement coaches or innovation mentors, tasking them to lead the specific knowledge‐sharing activities that our data show possess a strong effect size (OR = 2.568) to effectively uplift peers in the Moderate Innovation Profile (Gularte‐Rinaldo et al. 2023). This formal designation as ‘innovation champions’ is a proven strategy for diffusing innovation throughout an organization (Santos et al. 2022). To ensure this interaction thrives, managers must actively cultivate psychological safety, ensuring that ‘Moderate Innovation Profile’ feel secure in proposing nascent ideas without fear of failure, a critical factor for innovation in healthcare teams (O'Donovan and McAuliffe 2020). This structured peer‐to‐peer pairing is the key connector in the talent pipeline. In this process, nurses with high innovation profiles act as talent identifiers and transition facilitators, helping high‐potential peers with moderate innovation profiles bridge the gap from adaptive to proactive innovation. This integrated strategy, aimed at promoting upward mobility, truly leverages the identified workforce heterogeneity.

In conclusion, this study's person‐centred approach successfully revealed the heterogeneity of innovative behaviour in community nursing. The identification of the ‘Moderate Innovation Profile’ and ‘High Innovation Profile’ profiles moves beyond a simplistic linear view of innovation and provides a more nuanced understanding of the workforce. These findings provide a clear mandate for administrators to abandon one‐size‐fits‐all strategies by providing foundational training for the moderate group while utilizing the high group to drive peer learning.

## 5 Limitations

This study has several methodological limitations regarding its design, data collection procedures and statistical analysis that should be acknowledged. First, the cross‐sectional design prevents establishing definitive causal relationships, which introduces the potential impact of overestimating the long‐term stable predictive power of organizational and personal antecedents on profile membership. Second, the reliance on convenience sampling introduces a distinct self‐selection bias, where highly motivated or innovative nurses were likely more eager to participate, thereby potentially artificially inflating the true proportion of the High Innovation Profile. Regarding data collection procedures, utilizing an electronic survey with a strict three‐minute minimum completion threshold based on pre‐test results may have excluded rapid responders, creating an operational bias that limits the representation of certain response styles. Furthermore, because the sample was drawn exclusively from a single developed metropolitan network, regional and institutional clustering bias may restrict the generalizability of this two‐profile model to resource‐constrained or rural primary care settings. Third, all measures were self‐reported, making the findings prone to social desirability bias where participants might overreport proactive behaviours, potentially introducing measurement bias that artificially alters classification accuracy. Furthermore, the innovation scale captures only behavioural frequency rather than the actual clinical quality or objective organizational impact of those innovative efforts. Future research must link these self‐reported profiles with administrative records or supervisor‐rated outcomes to more thoroughly validate the practical relevance of the identified subgroups. Finally, specific to the latent profile analysis, utilizing a limited number of indicators based on aggregated dimension scores rather than item‐level data may have obscured subtle micro‐level variations within individual innovation sub‐domains. Moreover, the total lack of split‐sample validation or external cross‐validation means the structural stability and replicability of this final two‐profile solution across independent cohorts remain completely unverified. Additionally, regarding the regression analysis, the two step variable selection approach that screened predictors through univariate analysis may have omitted complex confounding relationships or inflated chance findings. Despite these limitations, this study establishes an empirical baseline for addressing innovation heterogeneity and designing targeted primary care workforce interventions.

## 6 Conclusion

This study indicates a level‐based heterogeneity within the sampled community nursing workforce, identifying two distinct sub‐groups: a Moderate Innovation Profile (50.4%) and a High Innovation Profile (49.6%). The original contribution of this research lies in demonstrating that innovation sub‐groups among community nurses differ primarily by cumulative behavioural volume rather than morphological style, suggesting that nursing managers should move beyond uniform approaches. Consequently, local nursing administrators are encouraged to consider these stratified characteristics when allocating development resources. Specifically, instead of implementing universal incentives, managers might provide foundational continuing education for the moderate group while utilizing the high group for peer‐mentorship roles to optimize local nursing innovation potential. Given the cross‐sectional nature of this design, future longitudinal research is necessary to track profile stability over time and integrate objective clinical outcomes to fully validate the long‐term effectiveness of these stratified strategies.

## 7 Relevance for Clinical Practice

Identifying distinct innovation profiles translates these empirical findings into a concrete foundation for evidence‐based community nursing management, strategic staffing, and primary healthcare policy implementation. By providing objective evidence on nurse innovation heterogeneity, this framework allows administrators to move away from intuitive decisions. Instead, managers can utilize these profile distributions to guide strategic staffing and targeted training, ensuring that frontline nursing practices, such as chronic disease management, are driven by robust empirical data. At the policy level, healthcare authorities should align continuing education mandates with digital readiness by institutionalizing telehealth competency training and establishing centralized knowledge‐sharing platforms across primary health networks.

For community nurses within the Moderate Innovation Profile, who represent the stabilization baseline of the primary care workforce, administrative decisions should focus on lowering boundaries and building foundational innovation confidence. Specifically, during routine clinical case audits or community health briefings, managers can actively guide these nurses to identify repetitive pain points in daily operations, such as community chronic disease management workflows or home‐based wound care protocols. Decision‐makers can then allocate small‐scale institutional funding, provide training or protected hours to support this group in executing incremental micro‐innovations, such as optimizing patient scheduling procedures or refining localized health‐education toolkits.

Conversely, for nurses belonging to the High Innovation Profile, the decision‐making process should pivot toward clinical autonomy and peer‐led knowledge translation. Managers should explicitly identify these high‐capacity individuals to serve as innovation champions or clinical mentors within community health centres. In practice, when designing complex, multi‐disciplinary primary care pathways or driving digital health integrations, administrators should grant this subgroup greater clinical autonomy, flexible working hours and direct representation in project planning. Tasking them with leading quality improvement initiatives and formal mentorship roles allows organizations to strategically leverage their advanced competency, ultimately transforming individual innovative capacity into system‐wide clinical value. To ensure effective future implementation, primary care networks must establish a dynamic talent development ladder rather than treating nurse profiles as static labels.

## Author Contributions

**Shengcai Zhu:** conceptualization, writing – original draft, data curation, visualization, methodology. **Jiaojiao Hu:** conceptualization, writing – original draft, methodology, investigation. **Beirong Mo:** data curation, writing – original draft. **Junze Yan:** resources, writing – original draft. **Kunqi Ma:** resources, data curation. **Yumei Liao:** investigation, data curation, resources. **Li Wang:** funding acquisition, writing – review and editing.

## Funding

This study was funded by the Shenzhen Science and Technology Innovation Commission Fund of 2023 (Grant number: JCYJ20230807115904009).

## Statistical Declaration

The statistical analyses in this study were performed using Mplus version 8.3 and SPSS version 27.0. All analyses, including Latent Profile Analysis, were conducted independently by two members of the research team and cross‐checked for consistency, in accordance with the STROBE reporting guidelines for observational studies.

## Ethics Statement

This study was conducted in accordance with the principles of the Declaration of Helsinki. Ethical approval was obtained from the Ethics Committee of Shenzhen Nanshan People's Hospital (Approval No. ky‐2025‐062301). All participants provided written informed consent before their inclusion in the study.

## Conflicts of Interest

The authors declare no conflicts of interest.

## Data Availability

The data that support the findings of this study are not publicly available due to the sensitive and personal nature of the interview transcripts but are available from the corresponding author upon reasonable request.
